# Cleavage of von Willebrand Factor by Granzyme M Destroys Its Factor VIII Binding Capacity

**DOI:** 10.1371/journal.pone.0024216

**Published:** 2011-09-01

**Authors:** Martine J. Hollestelle, Ka Wai Lai, Marcel van Deuren, Peter J. Lenting, Philip G. de Groot, Tom Sprong, Niels Bovenschen

**Affiliations:** 1 Department of Clinical Chemistry and Hematology, University Medical Center Utrecht, Utrecht, The Netherlands; 2 Department of Pathology, University Medical Center Utrecht, Utrecht, The Netherlands; 3 Department of Medicine and Nijmegen Institute for Infection, Inflammation and Immunity (N4i), Radboud University Nijmegen Medical Center, Nijmegen, The Netherlands; 4 INSERM U.770, Le Kremlin Bicetre, France; Emory University School of Medicine, United States of America

## Abstract

Von Willebrand factor (VWF) is a pro-hemostatic multimeric plasma protein that promotes platelet aggregation and stabilizes coagulation factor VIII (FVIII) in plasma. The metalloproteinase ADAMTS13 regulates the platelet aggregation function of VWF via proteolysis. Severe deficiency of ADAMTS13 is associated with thrombotic thrombocytopenic purpura, but does not always correlate with its clinical course. Therefore, other proteases could also be important in regulating VWF activity. In the present study, we demonstrate that VWF is cleaved by the cytotoxic lymphocyte granule component granzyme M (GrM). GrM cleaved both denaturated and soluble plasma-derived VWF after Leu at position 276 in the D3 domain. GrM is unique in that it did not affect the multimeric size and pro-hemostatic platelet aggregation ability of VWF, but instead destroyed the binding of VWF to FVIII *in vitro*. In meningococcal sepsis patients, we found increased plasma GrM levels that positively correlated with an increased plasma VWF/FVIII ratio *in vivo*. We conclude that, next to its intracellular role in triggering apoptosis, GrM also exists extracellularly in plasma where it could play a physiological role in controlling blood coagulation by determining plasma FVIII levels via proteolytic processing of its carrier VWF.

## Introduction

Von Willebrand factor (VWF) is a pro-hemostatic multimeric plasma protein that promotes platelet adhesion by bridging injured subendothelium and platelet receptors [1]. In addition, VWF tightly binds to and stabilizes coagulation factor VIII (FVIII) in plasma, thereby protecting FVIII from proteolytic degradation and prolonging FVIII half-life [2]. The multimeric structure of VWF is important for multiple interactions with platelets and endothelial components under high shear stress. Interaction with subendothelial structures induces a conformational change of VWF and the exposure of platelet binding domains [3]. This pro-hemostatic activity of VWF is regulated by the metalloprotease ADAMTS13, which specifically cleaves VWF in its A2 domain [4]. ADAMTS13 downregulates the multimeric size of VWF in order to prevent unwanted platelet thrombus formation. Deficiency of ADAMTS13 as is present in patients with thrombotic thrombocytopenic purpura (TTP) results in the occurrence of microvascular platelet thrombi in different organs, demonstrating its physiological importance [5]. However, low ADAMTS13 levels are also observed in TTP patients in remission [6], [7], not all patients with congenital ADAMTS13 deficiency develop TTP [8], and increased VWF proteolysis has been identified in acute TTP patients without loss of larger multimers [9]. These findings strongly suggest the existence of other important proteases that regulate VWF activity, in addition to ADAMTS13. Indeed, the VWF A2 domain has been identified as a hot spot for proteolysis by ADAMTS13 and other (leukocyte) proteases [10], [11]. Buzza *et al*. have shown that VWF activity *in vitro* could be regulated via cleavage by a serine protease from the granules of cytotoxic lymphocytes, *i.e.* granzyme B (GrB) [12].

GrB is secreted by cytotoxic T lymphocytes (CTLs) and natural killer (NK) cells, which are key players in eliminating tumor cells and pathogen-infected cells [13]. Besides GrB, four other granzymes are known in human, *i.e.* GrA, GrH, GrK, and GrM, which differ on the basis of their substrate specificity [13]. In addition to their well-established roles as intracellular effectors of target cell death, increasing evidence has been composed that granzymes may have extracellular roles as well. Elevated levels of circulating GrA, GrB, and GrK occur in certain allergic responses, rheumatoid arthritis, viral/bacterial infections, and sepsis [14]–[16]. GrB is also elevated in several vascular diseases such as arthrosclerosis, where both local and plasma levels correlate with disease severity [17], [18]. GrB does not cleave soluble VWF in plasma but cleavage is induced when VWF is unfolded or released from activated endothelial cells under physiological shear, thereby mimicking the action of ADAMTS13 to regulate the platelet-adhesive function of VWF [12]. Whether other granzymes than GrB also cleave VWF and affect VWF function remains unknown.

Whereas ADAMTS13 and GrB cleave VWF only under denaturating conditions or physiological shear stress, we demonstrate in the present study that GrM cleaves both denaturated and soluble plasma-derived VWF in its D3 domain. Unlike GrB, GrM did not affect the multimeric size and pro-hemostatic platelet aggregation ability of VWF, but instead destroyed the binding of VWF to FVIII *in vitro*. In meningococcal sepsis patients, we found increased plasma GrM levels that positively correlated with an increased plasma VWF/FVIII ratio *in vivo*. Our data indicate for the first time that extracellular GrM exists in plasma, where it could play a physiological role in controlling blood coagulation by determining plasma FVIII levels via proteolytic processing of its carrier VWF.

## Materials and Methods

### Ethics Statement

Informed consent was given for each patient by the parents and the study was approved by the local ethics committee and performed according to local guidelines.

### Materials

Plasma-derived human VWF was purified from VWF/FVIII concentrate as described. [19] Human placenta collagen type III was from Sigma. Recombinant human FVIII was obtained from Baxter (Advate). Ristocetin was from DiaMed (DiaRistin, Switzerland). The rabbit polyclonal anti-human VWF antibody was from DAKO. Active recombinant human granzymes (GrA, GrB, GrH, GrK and GrM) and inactive granzyme mutants (SA-variants) were expressed in *Pichia pastoris* and purified by cation-exchange chromatography as previously described [20], [21]. Granzymes were active as determined by small synthetic chromogenic substrates and their cleavage potential of macromolecular substrates (data not shown). Protein concentrations were measured by the method of Bradford.

### VWF cleavage assays

Cleavage of VWF was performed either in solution or coated onto a plastic plate. For immobilized VWF, purified plasma VWF (2 µg/well) in 100 µL of PBS was incubated overnight at 4°C in a Maxisorp 96-well plate (Nunc, Roskilde, Denmark). Wells were washed three times with PBS and treated with the various purified recombinant granzymes in 50 µL of Tris-buffered saline (TBS) (20 mM Tris, 150 mM NaCl, pH 7.4) for 1 hour at 37°C. VWF cleavage in solution was performed with indicated concentrations of VWF and granzymes in either TBS or 5 mM Tris-HCl, pH7.4. The incubations were stopped by adding reducing Laemmli sample buffer. Samples were resolved on 6% SDS-PAGE gels and transferred to a PVDF membrane (Millipore). The VWF cleavage products were visualized using a HRP-conjugated anti-VWF antibody (Dako Glostrup, Denmark). For determination of the GrM cleavage site, the VWF cleavage product was isolated form the PVDF membrane, which was stained with Coomassie Brilliant Blue. N-terminal sequencing (Edman degradation) was outsourced to Eurosequence (Groningen, The Netherlands).

### VWF multimer analysis

Purified plasma VWF (2 µg/mL) was incubated with indicated concentrations of GrM, GrB, GrM-SA, and GrB-SA in 5 mM Tris (pH7.4) for 1 hour at 37°C. VWF multimers were separated by electrophoresis using a 1% stacking and 2% running separating SDS agarose gel. Subsequently, proteins were transferred to a PVDF nylon membrane, and the multimers were detected using anti-human VWF peroxidase-labeled antibody (Dako). The peroxidase label was visualized by incubation with 0.5 mg/mL diamino benzidine, 0.3 mg/mL CoCl_2_, 0.2 mg/mL NiSO_4_ and 0.02% H_2_O_2_ in PBS.

VWF activation factor- Purified plasma VWF (2 µg/mL) was incubated with GrB, GrM, or buffer only in 5 mM Tris (pH7.4) at 37°C for 1 hour and active VWF was detected by immunosorbent assay. The VWF activation factor was determined by measuring the levels of activated VWF that recognizes the active, GPIb**α** binding conformation by using the AU/VWFa-11 llama-derived nanobody, as described by Hulstein *et al*. [22] The VWF activation factor was calculated by dividing the absorbance slope of a sample to the slope of the corresponding standard sample (normal plasma from a pool of more than 150 adult donors served as standard, also referred to as normal pool plasma, NPP) and correct them for VWF levels.

### VWF ristocetin cofactor assay

Purified plasma VWF (15 µg/mL) was incubated for 1 hour at 37°C with purified GrM, GrM-SA, or GrB in 5 mM Tris (pH7.4). VWF ristocetin cofactor activity (VWF:RCof) was determined using the BC von Willebrand reagent (Dade Behring Marburg GmbH, Marburg, Germany) on the BCS apparatus according to the manufacturer's instructions.

### Platelet aggregation assay

Blood from healthy donors was collected in 3.8% Tri-sodiumcitrate. The donors had not taken any aspirin or other non-steroidal anti-inflammatory drugs in the previous 2 weeks. Platelet-rich plasma (PRP) was prepared by centrifugation at 160 g for 15 min at room temperature. The washed platelets were prepared as described previously [23]. Washed platelet suspension was further diluted in HEPES-Tyrode buffer (pH7.3) to a platelet count of 200×10^9^/L (20,000/µL) and used in a ristocetin-induced aggregation assay. Purified VWF (6 µg/mL) was incubated with indicated concentration of GrB or GrM at 37°C for 1 hour. The incubation product (250 µL) was added to 250 µL of platelet suspension. Platelet aggregation was induced by adding 1.5 mg/mL ristocetin (DiaRistin, DiaMed, Switzerland).

### VWF-collagen binding assay

Purified VWF (1 µg/mL) was incubated with indicated concentration of GrB, GrB-SA, GrM or GrM-SA in 5 mM Tris buffer (pH 7.4) at 37°C for 1 hour. Subsequently, the incubation product was added to a well coated with collagen type III (Sigma) (0.1 mg/mL) in 100 µL PBS in a costar 96-wells plate by centrifugation. The VWF-collagen binding assay was carried out as described previously [24].

### VWF-FVIII binding assays

For immunosorbent assay, a 96 wells ELISA plate was coated with 10 µg/mL recombinant FVIII (Advate, Baxter AG, Austria) in 100 µL/well TBS, 10 mM CaCl_2_, pH 7.4, overnight at 4°C. The plate was washed three times (TBS, 0.1% Tween, 10 mM CaCl_2_) and blocked with TBS, 3% BSA, 0.1% Tween, 10 mM CaCl_2_ at 37°C for 1 hour. Meanwhile, purified plasma VWF (1 µg/mL) was pre-incubated with indicated concentration of GrB, GrB-SA, GrM or GrM-SA in 5 mM Tris, pH 7.4 at 37°C for 1 hour. Cleaved VWF (10 µL) was diluted 10× in TBS, 3% BSA, 0.1% Tween, 10 mM CaCl_2_ and added to the well for 2 hours at 37°C. After washing the plate three times with TBS, 0.1% Tween, 10 mM CaCl_2_, bound VWF was detected with a HRP-conjugated anti-VWF antibody for 30 minutes at 37°C. For immunoprecipitation analysis, VWF (30 ng) was pre-incubated in the presence or absence of GrM (100 nM) or GrM-SA (100 nM) in 5 mM Tris, pH7.4 for 1 hour at 37°C, followed by incubation with purified FVIII heterodimer (50 nM) (Advate, Baxter) in TBS, 10 mM CaCl_2_ for 2 hours at 37°C. Protein mixtures were subsequently incubated with ProtA/G sepharose (25 µL) (Thermo Scientific) in TBS, 10 mM CaCl_2_, which was pre-treated with anti-VWF antibody (300 µg) for 2 hours at room temperature and blocked in TBS, 10 mM CaCl_2_, 1% BSA for 30 minutes at room temperature. Samples were resolved in Laemmli sample buffer and subjected to SDS-PAGE (7.5%) and Western blotting, using polyclonal antibodies against VWF (Dako Glostrup, Denmark) and FVIII (Affinity Biologicals NC).

### Plasma measurements in patients with meningococcal disease

Between 2002 and 2008 from 37 patients with meningococcal disease were admitted to our pediatric intensive care unit (PICU). From 31 of these patients plasma was available for analysis. Informed consent was given for each patient by the parents and the study was approved by the local ethics committee and performed according to local guidelines. Diagnosis of meningococcal disease was made based on positive culture of blood, cerebrospinal fluid (CSF) or skin biopsy, or typical presentation. All isolated meningococci were group B. Septic shock was defined according to the consensus conference definitions for sepsis and organ dysfunction in pediatrics [25]. All patients received prompt antibiotic treatment (ceftriaxone) and dexamethasone (4×0.15 mg/kg) during the first 3 days after admission or until discharge from the PICU. Additional blood samples were collected approximately one year after acute disease from 5 patients that recovered and were defined in this study as healthy controls. Plasma samples were anti-coagulated with sodium citrate and were stored at −80°C prior to use. VWF antigen measurement and other clinical characteristics were described previously [16]. FVIII measurement was performed with Asserachrom kit® according to the manufacturer's instructions (Diagnostic Stage, Asnieres, France). Mouse anti-human GrM monoclonal antibody was raised as previously described. [26] Patient plasma samples (5 µL) were resolved on 12% SDS-PAGE gels and transferred to the PVDF membrane. GrM was detected by mouse anti-human GrM monoclonal antibody (clone 4A8E11, 1.0 µg/mL), followed by Goat anti-Mouse HRP-conjugated secondary antibody (Biosource, CA, US). Protein was visualized by enhanced chemiluminescence (ECL) (GE Healthcare, Buckinghamshire, UK), bands were quantified using a densitometer (ImageQuant TL, Amersham Bioscience), and quantified using a calibration curve of purified recombinant GrM.

### Statistical Analysis

All data were presented as mean and SD and compared using a T-test, unless otherwise stated. The Spearman correlation coefficient was calculated to determine the relationship between two parameters. A p-value <0.05 was considered to be statistically significant. The statistical analyses were performed with SPSS software, version 15.0.

## Results

### GrM cleaves VWF

**Figure 1:**
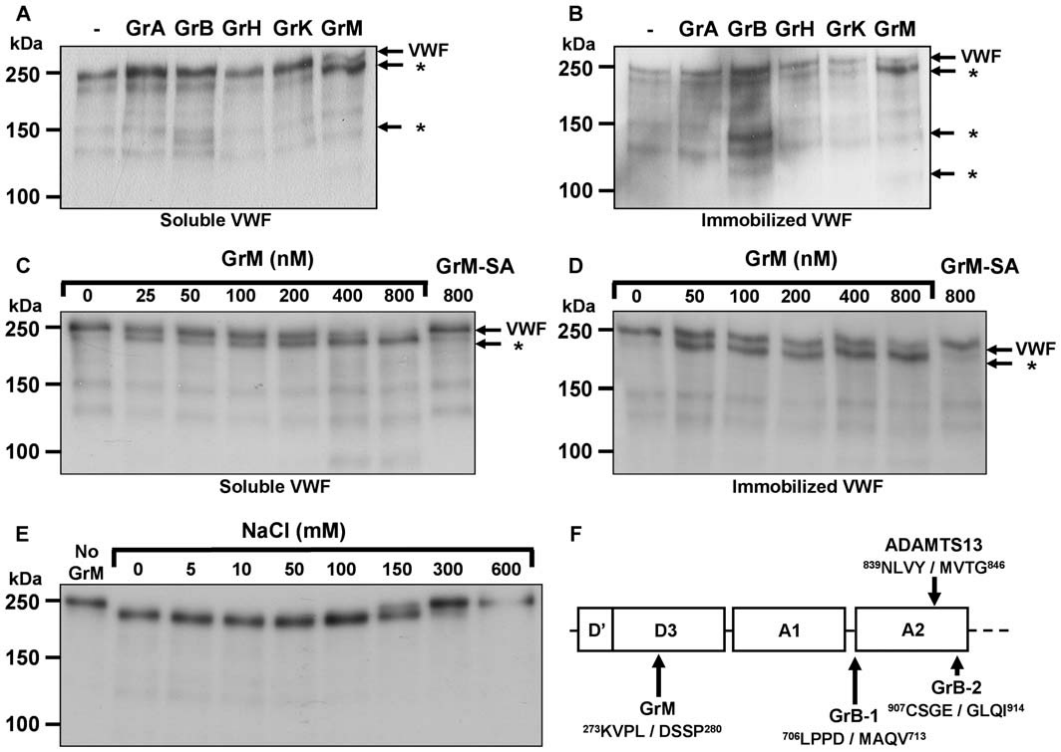
GrM cleaves VWF. (A) Purified plasma VWF (5 µg/mL) was incubated with various purified recombinant granzymes (300 nM) in TBS for 1 hour at 37°C and analyzed by immuno blot, using an antibody against VWF. Mature VWF and VWF cleavage fragments (*) are indicated by the arrows. (B) Purified plasma VWF (2 µg/well) was immobilized onto plastic and incubated with various purified recombinant granzymes (300 nM) in TBS for 1 hour at 37°C and analyzed by immuno blot, using an antibody against VWF. (C) Purified plasma VWF (5 µg/mL) was incubated with different concentrations of GrM (0–800 nM) or GrM-SA (800 nM) in TBS for 4 hours at 37°C and analyzed by immuno blot, using an antibody against VWF. (D) Purified plasma VWF (2 µg/well) was immobilized onto plastic and incubated with GrM (0–800 nM) or GrM-SA (800 nM) in TBS for 1 hour at 37°C and analyzed by immuno blot, using an antibody against VWF. (E) Purified plasma VWF (5 µg/mL) was incubated with GrM (100 nM) for 1 hour at 37°C in Tris (pH 7.4) in the presence or absence of increasing concentrations of NaCl (0–600 mM) and analyzed by immuno blot, using an antibody against VWF. (F) GrM, GrB, and ADAMTS13 cleavage sites in VWF are schematically indicated. Data are representative of at least three independent experiments.

ADAMTS13 and GrB cleave VWF only when the specific cleavage sites are exposed in the VWF A2 domain [27]–[29]. *In vitro* this denaturating conformational change can be induced by immobilization of VWF [12]. To investigate whether human granzymes other than GrB cleave VWF, we incubated all purified human granzymes with purified human plasma-derived VWF under soluble (Tris-buffered saline) (Fig. 1A) and immobilized conditions (Fig. 1B). As expected, GrB cleaved immobilized VWF more efficiently as compared with soluble VWF [12]. Interestingly, GrM cleaved both soluble and immobilized VWF with the concomitant appearance of a similar VWF cleavage fragment that differs from those induced by GrB (Fig. 1A,B). This suggests that GrM cleaves both soluble and immobilized VWF at the same site at a different position than GrB. GrA, GrH, and GrK did not cleave VWF under these conditions (Fig. 1A,B). All granzymes were catalytically active as determined by small synthetic substrates and by verifying cleavage of specific macromolecular granzyme substrates (data not shown). To further characterize VWF cleavage by GrM, purified VWF was incubated with different concentrations of GrM and the catalytically inactive GrM mutant (GrM-SA) as a control. GrM cleaved VWF in a concentration-dependent manner under both soluble (Fig. 1C) and immobilized (Fig. 1D) conditions, as illustrated by the progressive disappearance of the full-length VWF protein band (250 kDa) and the appearance of the specific 220 kDa cleavage fragment. GrM-SA did not cleave VWF, indicating that VWF cleavage by GrM fully depends on the catalytic activity of the protease (Fig. 1C,D). Another method to induce a conformational change of VWF *in vitro* is by removal of salt [28], [30]. The efficiency of VWF cleavage by GrM was inhibited by increasing the salt concentration and was improved by lowering the NaCl concentration, which coincided with the appearance of the 220 kDa VWF cleavage fragment (fig. 1E). Finally, N-terminal sequencing of this cleavage fragment revealed that GrM cleaved VWF in its D3 domain after Leu^276^, within the sequence ^273^KVPL↓DSSP^280^ (Fig. 1F).

### GrM does not affect VWF multimerization

**Figure 2:**
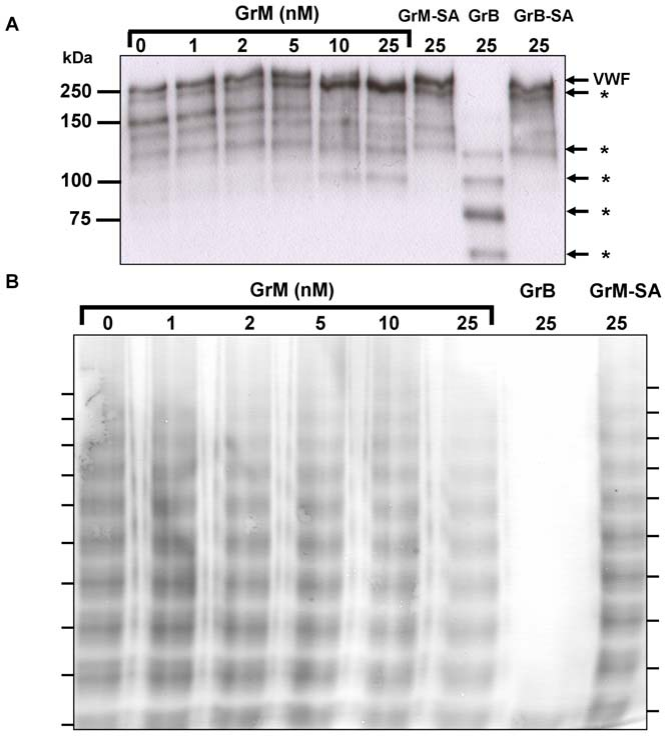
GrM does not affect VWF multimerization. Purified plasma VWF (2 µg/mL) was incubated with indicated concentrations of GrM, GrB, GrM-SA and GrB-SA in Tris (pH 7.4) for 1 hour at 37°C. (A) Samples analyzed at 6% SDS-PAGE and Western blot, using an antibody against VWF. Mature VWF and VWF cleavage fragments (*) are indicated by the arrows. (B) Samples were separated at a SDS agarose gel and multimers were visualized on blot. Data are representative of at least three independent experiments.

*In vivo*, all known VWF proteases regulate VWF activity by cleaving large multimers into less pro-hemostatic forms [6]. To investigate the effects of GrM on VWF multimerization pattern, purified VWF was incubated with several concentrations of GrM and VWF multimers were visualized by electrophoresis (Fig. 2). Whereas increasing concentrations of GrM gradually cleaved VWF (Fig. 2A), this cleavage did not significantly affect VWF multimerization, measured in four separated experiments (Fig. 2B). As a positive control, GrB cleaved VWF and showed a complete disappearance of the VWF multimerization pattern (Fig. 2). GrM-SA neither cleaved VWF nor affected VWF mutimer formation. These data indicate that unlike ADAMTS13 and GrB, no significant change in VWF multimerization is observed following treatment with GrM.

### GrM does not affect VWF platelet binding and platelet aggregation

**Figure 3 pone-0024216-g003:**
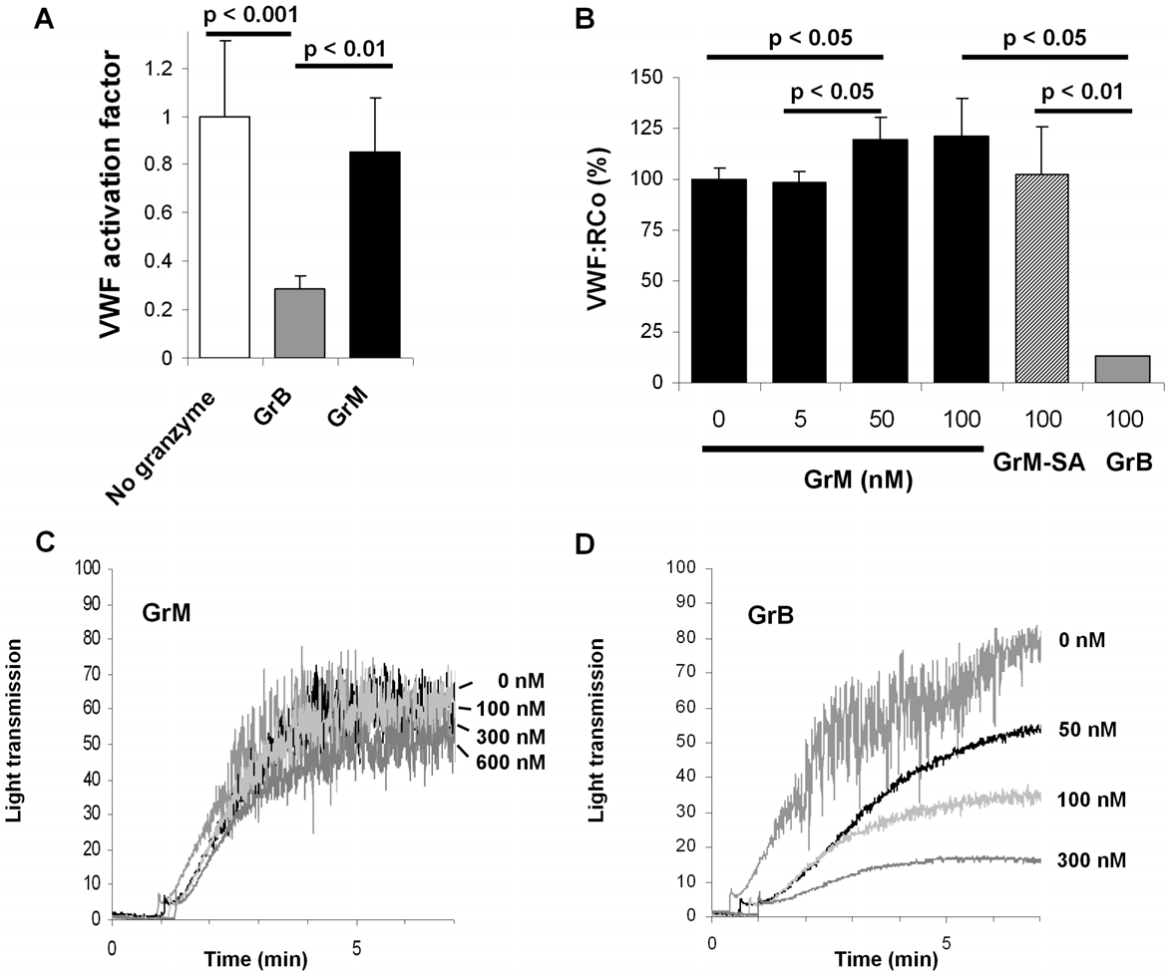
GrM does not affect VWF platelet binding affinity. (A) Purified plasma VWF (2 µg/mL) was incubated with GrB (100 nM), GrM (100 nM), or buffer only in Tris (pH 7.4) at 37°C for 1 hour and active VWF was detected by AU/VWF-A11-based immunosorbent assay. Data represent the mean +/− SD of three independent experiments. (B) Purified plasma VWF (15 µg/mL) was incubated for 1 hour at 37°C with various concentrations of GrM (0–100 nM), GrM-SA (100 nM), or GrB (100 nM) in Tris (pH 7.4) and VWF ristocetin cofactor activity was determined. Data represent the mean +/− SD of three independent experiments. (C) Purified plasma VWF (6 µg/mL) was treated with GrM (0–600 nM) or buffer only in Tris (pH 7.4) for 1 hour at 37°C and subsequently incubated 1∶1 with washed platelets (5×10^7^ platelets/mL). Platelet aggregation was induced by the addition of ristocetin (1.5 mg/mL). (D) Purified plasma VWF (6 µg/mL) was treated with GrB (0–600 nM) or buffer only in Tris (pH 7.4) for 1 hour at 37°C and subsequently incubated 1∶1 with washed platelets (5×10^7^ platelets/mL). Platelet aggregation was induced by the addition of ristocetin (1.5 mg/mL). Data are representative of at least four independent experiments with four different donors.

Another important characteristic of VWF is its binding to the GPIbα receptor expressed on platelets, thereby facilitating the binding of platelets to the injured vessel wall. First, with a specific assay using our AU/VWFa-11 llama-derived nanobody we are able to detect active VWF in its platelet-binding conformation [31], allowing us to determine which proportion of VWF is able to bind to GPIbα receptor. GrM and GrB were incubated with VWF and cleaved VWF was subsequently incubated with the AU/VWFa-11 antibody. This analysis showed that the VWF activation factor was not affected by GrM cleavage as compared to control, whereas GrB inhibited the VWF activation factor for about 70% (Fig. 3A). Second, incubation of VWF with various concentrations of GrM under similar conditions did not decrease the VWF ristocetin cofactor activity, whereas GrB-treated VWF was clearly affected (Fig. 3B). Instead, GrM slightly but statistically significantly increased VWF ristocetin cofactor activity. Finally, purified VWF was pretreated with various concentrations of GrB or GrM and subsequently incubated with washed isolated platelets to measure platelet aggregation. The aggregation curves of the GrM-pretreated VWF were similar to the VWF without added granzyme in multiple donors (Fig. 3C). As expected, GrB dose-dependently inhibited platelet aggregation (Fig. 3D). These data indicate that GrM neither affects VWF platelet binding nor platelet aggregation.

### GrM slightly affects VWF collagen binding activity

**Figure 4:**
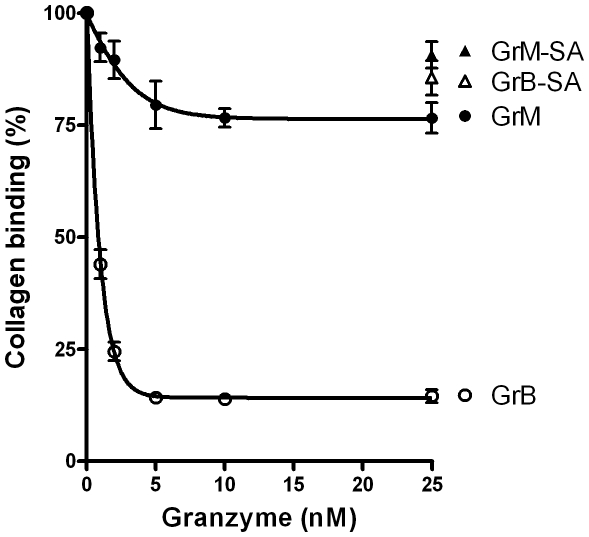
GrM slightly affects VWF binding to collagen. Purified plasma VWF (1 µg/mL) was incubated with indicated concentrations of GrB, GrB-SA, GrM, or GrM-SA in Tris (pH 7.4) for 1 hour at 37°C. Samples were assessed for collagen binding, using an immunosorbent assay. Data represent the mean +/− SD of three independent experiments. GrM (25 nM) versus GrM-SA (25 nM), p = 0.02; GrB (25 nM) versus GrB-SA (25 nM), p<0.001.

The effect of granzymes on collagen binding was investigated, using an immunosorbent assay. Collagen binding was almost fully abolished after treatment of VWF with GrB (Fig. 4) and following pre-incubation with the anti-VWF blocking antibody RU5 (data not shown). Pre-treatment of VWF with GrM resulted in a minor, but statistically significant, dose-dependent decrease in collagen binding as compared to control GrM-SA (Fig. 4, p = 0.02). Maximal decrease in collagen binding was achieved after treatment with 10 nM of GrM, where VWF was fully cleaved by GrM (Fig. 2A). These data indicate that GrM slightly affects VWF binding to collagen.

### GrM destroys the FVIII binding capacity of VWF

**Figure 5:**
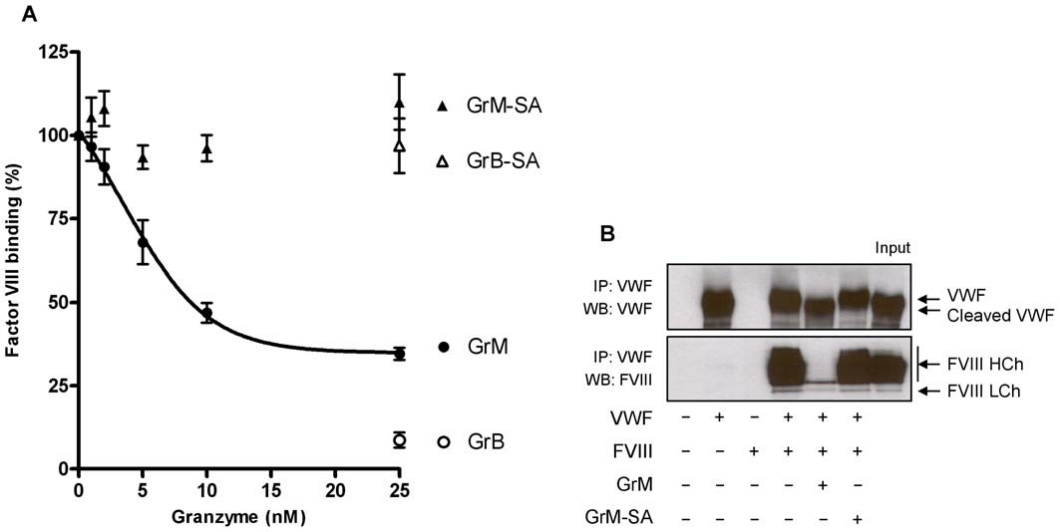
GrM disrupts FVIII binding to VWF. (A) Purified plasma VWF (1 µg/mL) VWF was incubated with indicated concentration of GrM, GrB, GrM-SA or GrB-SA in Tris (pH 7.4) for 1 hour at 37°C and subsequent FVIII binding was assessed in an ELISA setup. Data represent the mean +/− SD of three independent experiments. GrM (25 nM) versus GrM-SA (25 nM), p = 0.009; GrB (25 nM) versus GrB-SA (25 nM), p = 0.009. (B) VWF (30 ng) was pre-incubated in the presence or absence of GrM (100 nM) or GrM-SA (100 nM) in 5 mM Tris (pH 7.4) for 1 hour at 37°C, followed by incubation with purified FVIII heterodimer in TBS, 10 mM CaCl_2_ for 2 hours at 37°C. Protein mixtures were immunoprecipitated by ProtA/G sepharose pre-treated with anti-VWF antibody. Samples were analyzed by Western blotting, using antibodies against VWF and FVIII heterodimer. Full-length VWF, its GrM-cleaved form, and the FVIII heavy chain (HCh) and light chain (LCh) are indicated by the arrows. IP, immunoprecipitation; WB, Western blot. Data are representative of at least three independent experiments.

VWF tightly binds to and stabilizes coagulation FVIII in plasma, thereby protecting FVIIII from proteolytic degradation and prolonging FVIII half-life *in vivo* [2]. Using an immunosorbent assay, the FVIII binding capacity of VWF was investigated in the presence of GrM or GrB (Fig. 5A). A gradual decrease in FVIII binding up to about 70% was observed after treatment of VWF with increasing concentrations of GrM (Fig. 5A). Interestingly, GrB also inhibited VWF binding to FVIII. No effects were observed with both controls GrM-SA and GrB-SA. VWF cleavage by both granzymes was verified by immunoblot (Fig. 2A). The effect of GrM on VWF binding to FVIII was further investigated by immunoprecipitation analysis (Fig. 5B). Immunoprecipitation of VWF in the presence of FVIII resulted in the expected pull down of the FVIII heterodimer (Fig. 5B). In contrast, the VWF-FVIII interaction was almost completely abolished in the presence of GrM, but not in the presence of control GrM-SA (Fig. 5B). FVIII was not cleaved by GrM under these conditions (data not shown). These data indicate that both GrB and GrM destroy the FVIII binding capacity of VWF.

### Plasma GrM levels positively correlate with VWF/FVIII ratio in patients with meningococcal septic shock

**Figure 6:**
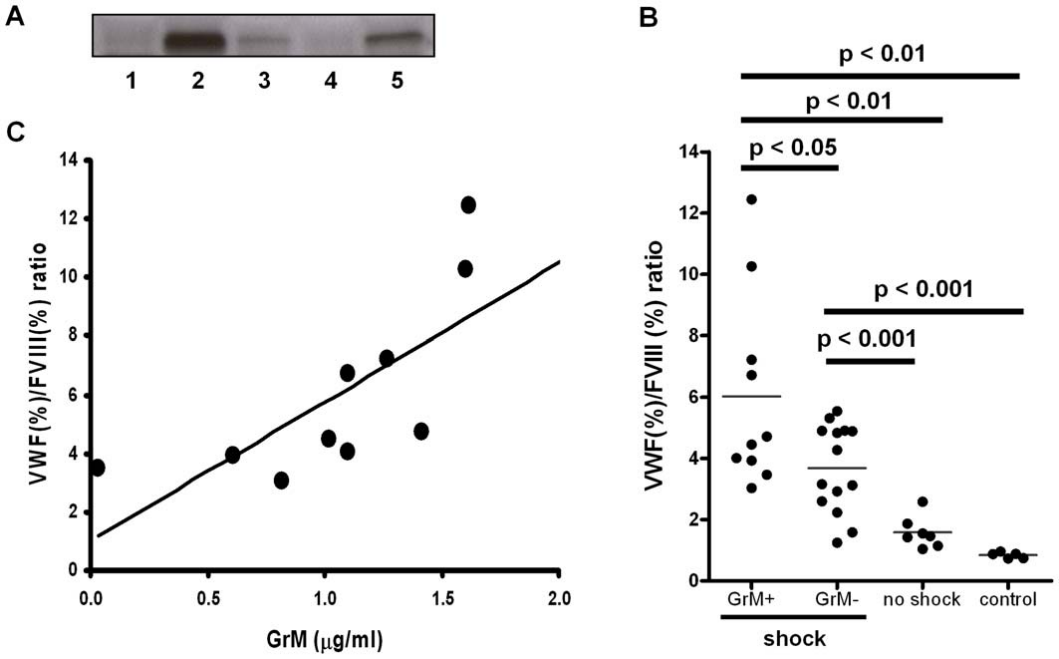
Plasma GrM and VWF/FVIII ratios positively correlate in patients with meningococcal disease. (A) Representative example of GrM in plasma of patients with meningococcal disease without shock (1) and with shock (2–5), using immunoblotting with a monoclonal antibody against GrM. (B) VWF and FVIII levels in plasma were determined by ELISA and chromogenic activity, respectively. VWF/FVIII ratios were determined in meningococcal shock patients with or without detectable plasma GrM, patients with meningococcal disease without shock, and healthy controls. P values are indicated. (C) VWF/FVIII ratios are plotted against plasma GrM levels, as quantified by western blot. R_spearman_ = 0.92, p<0.001.

If GrM cleaves VWF *in vivo* thereby decreasing the FVIII binding properties of VWF, one would expect an increased clearance of FVIII and a concomitant increase of the plasma VWF/FVIII ratio in the presence of GrM. Currently, it is unknown whether GrM exists in plasma of healthy subjects or under certain pathophysiological conditions. Since no quantitative measurements are available for detecting human GrM in plasma, we screened our recently developed panel of anti-human GrM monoclonal antibodies for reactivity on western blot [26]. Monoclonal antibody 4A8E11 worked well in this application and was specific for GrM, since it did not cross-react with the other human granzymes A, B, and K (data not shown). Previously, we have demonstrated that plasma levels of GrB are markedly increased in patients with meningococcal septic shock [16]. With our semi-quantitative western blot, we measured plasma levels of GrM in these patients and controls. No GrM could be detected in plasma of control patients and in patients without septic shock. Interestingly, in part of the meningococcal septic shock patients we could detect elevated levels of plasma GrM, which migrated at the expected molecular weight of 28 kDa (Fig. 6A). Next, we measured VWF and FVIII plasma levels and calculated the ratio between them. Normally, when VWF levels increase, FVIII levels increase to the same extent and would give a ratio of 1 (when both are expressed in percentage). In patients with septic shock and positive levels of GrM as quantified from western blot band intensities, the VWF/FVIII ratio was increased as compared to the other groups and controls (Fig. 6B, p<0.05). The VWF/FVIII ratio also correlated positive with the amount of GrM detected in GrM-positive meningococcal septic shock patients (R_spearman_ = 0.92, p<0.001, Fig. 6C). These data indicate that elevated plasma GrM is detected in meningococcal septic shock patients and that its levels positively correlate with the VWF/FVIII ratio, suggesting that GrM affects the FVIII binding capacity of VWF in these patients.

## Discussion

The granule-exocytosis pathway is the main mechanism by which cytotoxic lymphocytes kill target cells [13]. In human, five granzymes (GrA, GrB, GrH, GrK and GrM) are known to trigger cell death, of which GrA and GrB have been best studied. For GrA, GrB, and GrK, it has been demonstrated that they also exist extracellularly where they have been postulated to play a role in extracellular matrix degradation (GrB) [14], [15], [18], [32], [33] or pro-inflammatory cytokine release (GrA) [34]. In this study, we have shown for the first time that GrM also exists extracellularly in plasma of patients with meningococcal disease (Fig. 6). We found that GrM efficiently cleaves plasma-derived VWF. It has been well established that VWF activity *in vivo* is regulated by ADAMTS13 and potentially other proteases, including GrB and some leukocyte proteases [10], [12]. In contrast to these proteases that cleave VWF only when it is artificially unfolded or presented as a matrix, GrM cleaved plasma-derived VWF under both soluble and denatured conditions. GrM cleavage did not affect the multimeric size of VWF and no decrease in pro-hemostatic platelet aggregation ability of VWF was observed (Figs. 2–3). Instead, GrM is the first protease identified so far that cleaves VWF in its FVIII binding site, thereby specifically destroying the binding of VWF to FVIII *in vitro* (Fig. 5). In meningococcal septic patients, we found increased plasma GrM levels that positively correlated with an increased plasma VWF/FVIII ratio *in vivo* (Fig. 6). Our data indicate that, next to its intracellular role in triggering apoptosis, extracellular GrM could play a physiological role in controlling blood coagulation by determining plasma FVIII levels via proteolytic processing of its carrier VWF. Together with previous findings that GrB cleaves fibrinogen and VWF thereby helping to control localized coagulation [12], our results point to an important cross-talk between immune and coagulation systems.

Many proteases have been identified that cleave VWF within or near a hotspot region in its A2 domain, including ADAMTS13, GrB, and several leukocyte proteases [10], [12], [35]. Interestingly, GrM cleaved VWF outside this hotspot region in the D3 domain, thereby removing a relatively small N-terminal region from the mature VWF molecule. GrM cleavage occurred after P1-Leu at position 276 in the sequence Lys-Val-Pro-Leu-↓-Asp-Ser-Ser-Pro. These P1 (*i.e.*, Leu) and P2–P4 subsite amino acids (*i.e.*, Lys-Val-Pro) are completely consistent with previous advanced positional scanning of GrM substrate specificity with tetra-peptide libraries (PS-SCL) [36]. This analysis has revealed that Lys-Val-Pro-Leu is the most optimal P4-P1 sequence for human GrM cleavage. Since the pro-hemostatic activity of VWF is largely dependent on regions outside the D3 domain, one would expect that GrM-cleaved VWF retains its pro-hemostatic activity. Indeed, although GrM slightly affected VWF-collagen binding, GrM did not affect VWF multimerization or platelet binding and platelet aggregation (Figs. 2–4). Interestingly, VWF:RCo activity was slightly, but statistically significantly, elevated following GrM treatment (Fig. 3B). This is in agreement with a previous observation showing that removal of the VWF amino acids 1–272 increases the affinity of VWF for the GPIbα receptor [37]. We did not observe this effect in the platelet aggregation assay, but this could be due to a lower sensitivity of this assay. Cleavage after Leu at position 276 by GrM almost fully destroyed the ability of VWF to bind to FVIII (Fig. 5). This is compatible with an important FVIII binding site in the VWF D3 domain [38], [39]. GrB cleaves VWF in the A2 domain hotspot and not only abolishes VWF multimerization and platelet aggregation, but also impaired FVIII binding. Whether VWF-cleaving proteases other than GrM and GrB affect the VWF-FVIII interaction remains unknown.

The study of the physiological relevance of VWF-cleaving proteases *in vivo* is hampered by the lack of adequate mouse models. First, ADAMTS13-deficient mice do not develop TTP, indicating interspecies differences in VWF regulation [40]. Second, mouse granzymes display different substrate specificities than human granzymes [41], [42]. Finally, the GrM cleavage site (KVPL) is not conserved in mouse VWF and purified recombinant mouse GrM does not cleave mouse VWF in plasma (unpublished results). Therefore, we studied the physiological relevance of GrM in VWF biology in humans. As we have previously described for GrB [16], we demonstrated in the current study that also GrM is elevated in patients with septic shock (Fig. 6). Since GrB does not cleave VWF in its soluble conformation [12], it seems likely that if granzymes cleave VWF in plasma this will be mediated by GrM. However, it remains a question whether GrM levels in plasma are sufficient to cleave VWF *in vivo*. The exact GrM concentration in plasma could not be determined because no GrM ELISA is currently available. Since GrM is elevated in septic shock patients and abolishes VWF binding to FVIII, one would expect an increase in the VWF/FVIII ratio in these patients if GrM cleaves VWF *in vivo*. Indeed, an increase of the VWF/FVIII ratio was observed in septic shock patients that positively correlated with the amount of plasma GrM (Fig. 6). Although the direct relationship thereof remains to be determined, this is compatible with our hypothesis that GrM in plasma cleaves VWF and thereby negatively influences plasma levels of FVIII. Direct evidence for the cleavage of VWF by GrM and the concomitant regulation of FVIII levels in human plasma, however, will require a VWF mutant that is resistant to GrM proteolysis. Currently, this is not possible since there is no human patient known with a mutation in VWF at the GrM-cleavage site and also no human GrM-deficiencies have been described.

Modulation of granzyme activity *in vivo* could potentially have therapeutic implications. Increased consumption of active VWF together with platelets causes disseminated intravascular coagulation and subsequently bleeding complications, as we also have observed in our meningococcal patients [16]. Because GrB cleaves and destroys VWF pro-hemostatic activity *in vitro* [12], [16], this study], it will be of future interest to study whether GrB also inactivates VWF *in vivo* and thereby can be used as replacement for ADAMTS13 in diseases like TTP and/or during infection (where also lower ADAMTS13 levels are observed). Indeed, a first response by the body is to increase GrB levels in meningococcal patients [16], [43], but probably this is not enough to stop the massive coagulation activation in these patients by itself. Since GrM specifically targets the FVIII-binding capacity of VWF (Fig. 5), therapeutic administration of GrM concentrates in plasma may potentially lower plasma FVIII levels in patients with high FVIII and a concomitant tendency for developing thrombosis, while leaving the pro-hemostatic function of VWF intact. On the other hand, specific synthetic granzyme inhibitors could be a promising intervention strategy against bleeding complications in pathological disorders in which plasma granzymes are markedly elevated.
